# Cranberries

**Published:** 1870-10

**Authors:** 


### CRANBERRIES.

The acid of the cranberry is so decidely beneficial in all billious affections, by its stimulating effects upon the liver, that attention to its culture should be encouraged. One acre of land suitably prepared will yield two hundred and fifty dollars worth of cranberries; there are thousands of neglected acres of such land in New Jersey which are admirably adapted to this culture.

The cranberry plant is a beautiful evergreen, and grows thriftily; it can be kept all winter, and may be so trained to grow from flower-pots as to be beautifully ornamental to the parlor and dining-room through all seasons of the year. They will grow in any ordinary room, without special attention, and the berry will remain on the stem until the flowering for another crop. It flourishes farther north than any other berry, ripening on Bushman Island, on the western slope of Greenland, in latitude seventy-six.

